# Patterns and drivers of soil surface‐dwelling Oribatida diversity along an altitudinal gradient on the Changbai Mountain, China

**DOI:** 10.1002/ece3.10105

**Published:** 2023-05-19

**Authors:** Yiling Lin, Haitao Wu, Dong Liu, Yaxiao Li, Yujuan Kang, Zhongsheng Zhang, Wenfeng Wang

**Affiliations:** ^1^ Key Laboratory of Wetland Ecology and Environment, Institute of Northeast Geography and Agroecology Chinese Academy of Sciences Changchun China; ^2^ State Key Laboratory of Black Soils Conservation and Utilization, Northeast Institute of Geography and Agroecology Chinese Academy of Sciences Changchun China; ^3^ University of Chinese Academy of Sciences Beijing China

**Keywords:** biodiversity, ecological drivers, montane ecosystems, soil fauna, turnover

## Abstract

Distribution patterns of biodiversity and environmental interactions are dominant themes in ecology. In montane ecosystems, biodiversity is closely associated with altitudinal gradients. However, studies of biodiversity in montane ecosystems are focused on plants and vertebrates, with relatively less on invertebrates. Here, the present study used a Vortis arthropod suction sampler to explore the biodiversity patterns of soil surface‐dwelling Oribatida and their drivers along an altitudinal gradient (600, 800, 1600, 2000, and 2300 m) from typical temperate forests, evergreen coniferous forests, subalpine birch forests to alpine tundra on the north slope of Changbai Mountain, Northeast China. *Trichoribates berlesei*, *Platynothrus peltifer*, and *Oribatula tibialis* were the dominant soil surface‐dwelling species on Changbai Mountain. Generally, alpha diversity and beta diversity of soil surface‐dwelling Oribatida decreased with the rising altitude, with a peaking density value at 2000 m. The result of beta diversity showed that the structures of community were more influenced by the species turnover component than the nestedness component. Nonmetric multidimensional scaling (NMDS) ordination showed that the community structure of soil surface‐dwelling Oribatida varied significantly along the altitudinal gradient. The variance partitioning showed that the elevation and climatic conditions determined the soil surface‐dwelling Oribatida community. Spatial filtering represented by geographic and elevation distances was particularly associated with soil surface‐dwelling Oribatida community variation between altitudes on Changbai Mountain. However, the variation of the Oribatida community between adjacent altitudes was only associated with geographic distance. Our study provides supportive evidence for the biodiversity analyzing of soil surface‐dwelling Oribatida in montane ecosystems along an altitudinal gradient.

## Graphical abstract

The beta diversity of soil surface‐dwelling Oribatida was decreased with the altitude rising, and the structure of the community was more influenced by species turnover component than nestedness component.

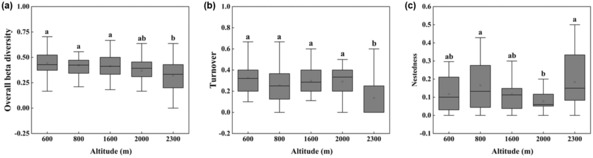

## 1 INTRODUCTION

Montane ecosystems comprise approximately 25% of the biodiversity of all terrestrial ecosystems (Miller & Spoolman, 2011; Rahbek et al., 2019). The most significant difference between montane and other ecosystems is the divergence in vertical zonation along the altitudinal gradient. The altitudinal gradient can be regarded similar to the climatic gradient, compressing the numerical variety of important factors such as temperature, precipitation, ultraviolet radiation, air pressure, wind velocity, and habitat characteristics into a relatively short geographical distance (Barry, 1992; Mayor et al., 2017; Reusch & Wood, 2007; Sundqvist et al., 2013). The altitudinal gradient significantly affects montane base resource availability and turnover efficiency (Gentili et al., 2013; Odland, 2009). Montane ecosystems provide a unique opportunity to study patterns and drivers of biodiversity along the altitudinal gradient.

Currently, studies of biodiversity patterns along the altitudinal gradient focus mainly on alpha diversity and less on beta diversity (Li et al., 2019; Winkler et al., 2018). Beta diversity can reflect the variation of biological communities along the habitat gradient (Bishop et al., 2015). Species turnover and nestedness are fundamental components of spatial community dissimilarity to measure the difference in species composition, species turnover replaces an existing species with a different species at a new site, and nestedness is the extent of species‐poor assemblages that constitute subsets of species‐rich assemblages (García‐Navas et al., 2020; Koleff et al., 2003; Wang et al., 2018). Thus, combining alpha diversity and beta diversity will support a better understanding of the biological distribution patterns of montane ecosystems, as well as predict the variation of the biological community to a changing environment (Gaston, 2000; Sundqvist et al., 2013).

Because of the workload involved in sampling invertebrates of montane ecosystems is enormous and complex, studies are often less focused on the variation pattern of invertebrates as compared to those for plants and vertebrates (Freeman, 2020; Sharma et al., 2019). Surface litter layer is a critical component of ecosystems and researchers have increasingly recognized the importance of the surface litter layer in recent years. The physical factors at the soil surface above the litter layers show more fluctuations than the soil environment (Otto & Svensson, 1982). Compared to soil invertebrates, soil surface‐dwelling invertebrates are closely related to plant diversity, and the variety of litter provides suitable microhabitats for soil surface‐dwelling invertebrates (Hasegawa et al., 2006; Xu et al., 2020). The high rate of litter turnover along an altitudinal gradient affects the exposure risk and food availability for invertebrates (Olson, 1994; Valencia‐Cuevas et al., 2020).

Oribatida are the most abundant microarthropods in forest ecosystems (Illig et al., 2010). They can directly accelerate organic matter decomposition, nutrient cycling through digestion and decomposing litter and play a critical role in soil microstructure formation (Bradford et al., 2002; González & Seastedt, 2001; Huhta, 2007; Lussenhop & Bassirirad, 2005; Wu et al., 2015; Yin et al., 2019). The diversity of Oribatida is influenced by a long‐term combination of factors (Mumladze et al., 2015). The inconsistent patterns of Oribatida along an altitudinal gradient are subject to environmental factors, geographical distance, and specific species characteristics (Illig et al., 2010; Marian et al., 2018). Influenced by these factors, the altitudinal diversity patterns of Oribatida generally decline linearly (Mumladze et al., 2017) or peak at an intermediate altitude, where the primary productivity is the highest (Liu et al., 2023).

The Changbai Mountain with its peak of 2691 m is the tallest in Northeast China with a significant vertical zonation. The vertical vegetation distribution on Changbai reflects the horizontal vegetation from temperate to boreal zones in Eurasia (Shao, 2011). This study addresses two questions, including: (1) what is the pattern of soil surface‐dwelling Oribatida diversity along an altitudinal gradient on Changbai Mountain? and (2) what are the drivers of the variation between different soil surface‐dwelling Oribatida assemblages? To study these research questions, we investigated soil surface‐dwelling Oribatida from 600 to 2300 m on Changbai Mountain. We hypothesize that (1) the distribution pattern of soil surface‐dwelling Oribatida is affected by deterministic and stochastic factors (Mumladze et al., 2015). Therefore, alpha diversity and beta diversity of soil surface‐dwelling Oribatida community would decrease with rising altitude, with a peak of diversity at mid‐altitudes. (2) Terrestrial invertebrates typically have a narrow altitudinal range of distribution (Brühl et al., 1999). Therefore, species turnover will be the main process of shaping the community structure of soil surface‐dwelling Oribatida along the altitudinal gradient. (3) The process of community construction related to environmental filtering and spatial filtering is essential to the dynamics of species distribution patterns (Heino et al., 2015; Peters et al., 2016), montane ecosystems provide variable environmental gradients, geographic distances, and elevation distances, we hypothesize that the variation in soil surface‐dwelling Oribatida community in different altitudes would be more related to spatial filtering and climate change driven by altitude variation rather than other environmental factors.

## 2 MATERIALS AND METHODS

### 2.1 Study area

This study was conducted on the Changbai Mountain Nature Reserve (41°41′49″–42°25′18″N, 127°42′55″–128°16′48″E), Northeast China. The region is one of the few well‐protected natural ecosystems (Shen et al., 2013). The region is characterized by a temperate continental mountain climate with a mean annual temperature of 3.6°C. The mean annual precipitation is 700 mm, with more than 80% of the precipitation occurring between June and September (Chen et al., 2011; Wu et al., 2002; Yu et al., 2013). Various distinctive vegetation types are found from the base to the summit of the mountain with the rising altitude. The typical temperate forests are observed below 1100 m (dominant tree species: *Pinus koraiensis*, *Quercus mongolica*, *Acer mono*, *Tilia amurensis*), the evergreen coniferous forests are between 1100 and 1700 m (dominant tree species: *Picea jezoensis*, *Abies nephrolepis*, *P. koraiensis*, *Betula costata*), the subalpine birch forests are between 1700 and 2000 m (dominant tree species: *Betula ermanii*, *Rhododendron aureum*), and the alpine tundra is above 2000 m (dominant tree species: *R. aureum*, *Trisetum spicatium*, *Vaccinium uliginosum*, *Rhododendron redovoskianum*, *Dryas octopetala*) (Shen et al., 2013).

### 2.2 Sampling methodology

**FIGURE 1:**
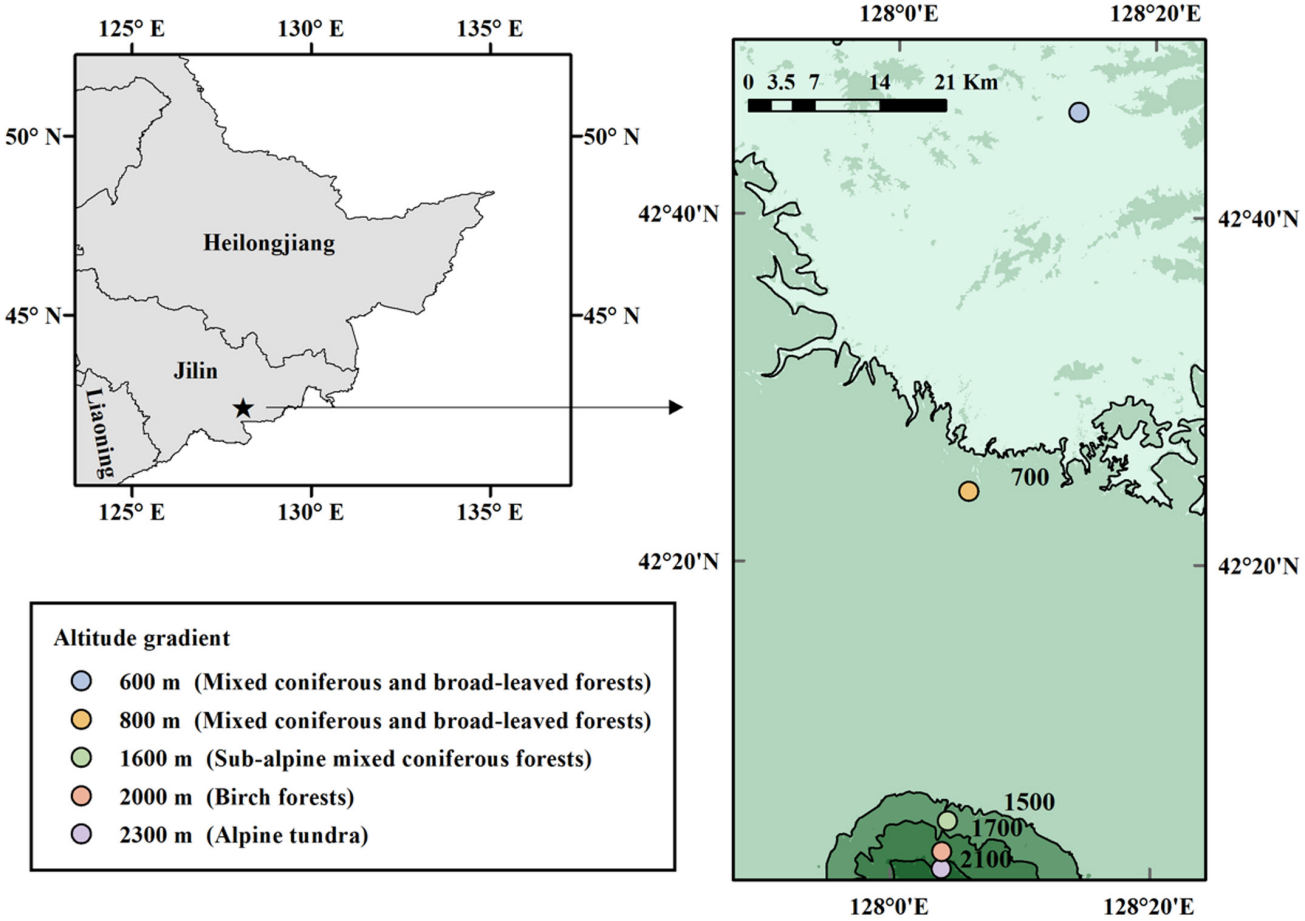
Geographic locations of the sampling sites along an altitudinal gradient on the Changbai Mountain, Northeast China.

We investigated soil surface‐dwelling Oribatida assemblages at five altitudes: 600 m (a mixed coniferous and broad‐leaved forest), 800 m (a mixed coniferous and broad‐leaved forest), 1600 m (a subalpine mixed coniferous forest), 2000 m (a birch forest), and 2300 m (an alpine tundra), along the Changbai Mountain in August 2021 (Figure 1). We selected nine plots at each altitude (600, 800, 1600, and 2000 m) except for the tundra zone. Because of the complex and diverse microhabitats, fifteen plots were selected in the tundra zone (2300 m). Each of the plots was separated by at least 50 m. Soil surface‐dwelling Oribatida were sampled with the Vortis arthropod suction sampler (Burkard Manufacturing Company Ltd, Rickmansworth, UK; within a 10 cm sampling radius and 1.0‐min sampling times at each plot). The sampler can filter extraneous materials, shorten the sampling periods, and is a standard suction sampler in scientific research for invertebrates such as Collembola, Acari, Araneae, and others. (Bohan et al., 2011; Fitzgerald & Solomon, 2004; Stewart, 2002; Zentane et al., 2016). The soil surface‐dwelling fauna was preserved in 95% alcohol and transported to the laboratory for identification. After submerging the Oribatida into lactic acid to make them fade, we identified and counted them under a stereo stereomicroscope with 6.3 to 80× magnification (SMZ1270, Nikon) (Niedbała & Liu, 2018; Ryabinin et al., 2018; Yin, 1998).

The coordinates of latitude, longitude, and elevation were recorded. Three litter samples of 1 m^2^ were mixed and collected at each altitude for testing the quality of litter per unit area (QUA), total carbon (TC), total nitrogen (TN), total hydrogen (TH), total sulfur (TS), and total phosphorus (TP). We dried the litter at 60°C for 48 h to measure QUA using an electronic scale (QUINTIX224‐1CN). TC, TN, TH, and TS were determined with an element analyzer (Carlo Erba FLASHEA 1112 CHN‐S analyzer) and TP with a continuous flow analyzer (SKALAR SAN++, SKALAR, Netherlands). Detailed physical and chemical information, sampling site coordinates, and elevation are described in Table 1. We used WorldClim version 2 to download the climate data (Fick & Hijmans, 2017) and extract the mean annual temperature (MAT) and mean annual precipitation (MAP) data by latitude and longitude coordinates.

**TABLE 1:** The mean value of environmental factors and coordinates of sampling sites along an altitudinal gradient on the Changbai Mountain.

	600 m	800 m	1600 m	2000 m	2300 m
TC (g/kg)	247.23	300.94	412.06	297.42	217.31
TN (g/kg)	11.85	14.31	16.12	12.92	8.87
TH (g/kg)	28.29	35.78	44.05	34.44	25.77
TS (g/kg)	0.37	0.37	0.60	0.41	0.18
TP (g/kg)	1.51	1.41	1.21	0.96	0.66
QUA (g/cm^2^)	0.11	0.20	0.63	0.51	0.58
MAT (°C)	3.23	2.81	−1.17	−3.13	−4.17
MAP (mm)	637	688	802	916	955
ALT (m)	600.00	775	1576.33	1988.67	2285.00
LAT	128.234139	128.096511	128.073096	128.066663	128.065722
LON	42.767008	42.402870	42.086343	42.057200	42.040437

Abbreviations: ALT, altitude; LAT, latitude; LON, longitude; MAP, mean annual precipitation; MAT, mean annual temperature; QUA, quality of litter per unit area; TC, total carbon; TH, total hydrogen; TN, total nitrogen; TP, total phosphorus; TS, total sulfur.

### 2.3 Statistical analysis

Based on the sampling radius of the Vortis arthropod suction sampler and species abundance, the species density of 1 m^2^ was calculated. Based on the abundance data, the soil surface‐dwelling Oribatida were classified into dominant species (>10% of the total number), common species (1–10% of the total number), and rare species (<1% of the total number) (Tan et al., 2010).

To explore altitudinal alpha diversity patterns of Oribatida assemblages, richness, Shannon–Weiner index (*H′*), and Simpson index (*λ*) were calculated (Shannon, 1948; Simpson, 1949). After testing the normality of the data, we chose the Kruskal–Wallis test to detect the difference in soil surface‐dwelling Oribatida diversity between different altitudes. Multiple linear regression was used to analyze the relationship between the density and alpha diversity of soil surface‐dwelling Oribatida community and altitude. Alpha diversity and multiple linear regression were calculated using the “diversity” and “lm” functions. The alpha diversity was calculated with the “vegan” package (Oksanen et al., 2013).

To assess differences in the Oribatida assemblage beta diversity pattern along the altitudinal gradient, we calculated pairwise beta diversity using the Sørensen dissimilarity index. Three matrices (Sørensen pairwise dissimilarity, *β*_sor_; Simpson pairwise dissimilarity, *β*_sim_; Nestedness‐resultant dissimilarity, *β*_sne_) of each altitude were constructed. The *β*_sor_ is overall beta diversity, *β*_sim_ and *β*_sne_ are turnover and nestedness components, respectively, and pairwise beta diversity was calculated with the “beta.pair” function in the “betapart” package (Baselga, 2010).

To visualize the differences between soil surface‐dwelling Oribatida community compositions along the altitudinal gradient, we calculated the non‐metric multidimensional scaling (NMDS) ordination based on Bray–Curtis dissimilarity matrix and analysis of similarity (ANOSIM) to test the significant differences between altitude gradients. NMDS and ANOSIM tests were calculated using the “metaMDS” and “anosim” functions. The “indicspecies” package was used to investigate the association between species and altitude by identifying the indicator soil surface‐dwelling Oribatida species of each altitude (Cáceres & Legendre, 2009; Dufrêne & Legendre, 1997).

After excluding the rare species (abundance <1%), variation partitioning analysis (VPA) was performed to explore the effect of environmental factors (including TC, TN, TP, TH, TS, and QUA), climate factors (including MAT and MAP), and an elevation factor (ALT) on variations of soil surface‐dwelling Oribatida community. The VPA was calculated with the “varpart” function in the “vegan” package, then used the “rda” function to test the significance of VPA results. We tested the correlation of spatial factors (including geographic distance and elevation distance) and soil surface‐dwelling Oribatida community using the Mantel analysis (Legendre & Legendre, 2012). The Mantel test was calculated with the “mantel” function in the “vegan” package. The geographic distance between each altitude was calculated with the “distm” function in the “geosphere” package (Hijmans et al., 2019). In addition, we used the ArcGIS 10.2 software to plot the study area and sampling sites. All data analyses were performed in R 4.2.0 (R Core Team, 2022).

## 3 RESULTS

### 3.1 Alpha diversity

A total of 34 species of soil surface‐dwelling Oribatida were identified along the altitudinal gradient (Table 2). Among them, *Trichoribates berlesei* (29.03%), *Platynothrus peltifer* (21.94%), and *Oribatula tibialis* (13.25%) were the dominant species, and *P. peltifer* was the dominant species in all the altitudes. Common species (13 taxa) and rare species (18 taxa) accounted for 28.99% and 6.78% of the total species abundance, respectively. *Cepheus cepheiformis*, *Heminothrus yamasakii*, and *Hermanniella granulata* were unique only in a mixed coniferous and broad‐leaved forest (600 and 800 m).

**TABLE 2:** The relative abundance of the soil surface‐dwelling Oribatida community along five altitudinal gradients in the Changbai Mountain, Northeast China; +++, dominant species; ++, common species; +, rare species.

Family	Species	600 m	800 m	1600 m	2000 m	2300 m
Achipteriidae Thor, 1929	*Achipteria coleoptrata* (Linnaeus, 1758)	++	++	++	+	++
Cepheusidae Berlese, 1896	*Cepheus cepheiformis* (Nicolet, 1855)	++	+		+	
Ceratoppiidae Grandjean, 1954	*Ceratoppia bipilis* (Hermann, 1804)	++	++	++		
Ceratozetidae Jacot, 1925	*Trichoribates berlesei* (Jacot, 1929)	++	++	+	+++	+++
Crotoniidae Thorell, 1876	*Heminothrus yamasakii* (Aoki, 1958)	+	+			
	*Platynothrus peltifer* (Koch, 1839)	+++	+++	+++	+++	+++
Cymbaeremaeidae Sellnick, 1928	*Scapheremaeus polysetosus* (Sitnikova, 1975)		+			
Damaeidae Berlese, 1896	*Dyobelba biclavata* (Wang & Norton, 1993)	++	++	+		
	*Epidamaeus* sp. nov.	+	++	++		+
	*Porobelba spinosa* (Sellnick, 1920)	+	+	++	++	
	*Tokukobelba compta* (Kulczynski, 1902)	++	++	++	+	+
Eniochthoniidae Grandjean, 1947	*Eniochthonius minutissimus* (Berlese, 1903)	++	++	+		
Eremaeidae Oudemans, 1900	*Eremaeus borealis* (Wen, 1988)	+	+	+	++	
Euphthiracaridae Jacot, 1930	*Acrotritia hauseri* (Mahunka, 1991)	+	++	+		+
Galumnidae Jacot, 1925	*Acrogalumna* sp. nov.	+	++		+	
Gustaviidae Oudemans, 1900	*Gustavia microcephala* (Nicolet, 1855)		++			
Hermanniellidae Grandjean, 1934	*Hermanniella granulata* (Nicolet, 1855)	+	+			
Humerobatidae Grandjean, 1971	*Diapterobates humeralis* (Hermann, 1804)	+				
Hypochthoniidae Berlese, 1910	*Hypochthonius rufulus* (Koch, 1835)	++	++			
Malaconothridae Berlese, 1916	*Malaconothrus pygmaeus* (Aoki, 1969)	+	++	++		
Mesoplophoridae Ewing, 1917	*Archoplophora rostralis* (Willmann, 1930)	+	++			+
Micreremidae Grandjean, 1954	*Micreremus brevipes* (Michael, 1888)	+	+	++		
Nanhermanniidae Sellnick, 1928	*Nanhermannia nana* (Nicolet, 1855)	+	+			
Oppiidae Sellnick, 1937	*Lauroppia neerlandica* (Oudemans, 1900)	++	++	++	+	++
Oribatulidae Thor, 1929	*Oribatula tibialis* (Nicolet, 1855)	+++			+	
Oribotritiidae Balogh, 1943	*Oribotritia gigas* (Bayoumi et Mahunka, 1979)	+	+	+		+
Phenopelopidae Petrunkevitch, 1955	*Eupelops contaminatus* (Choi, 1986)	++				
Protoribatidae Balogh et P. Balogh, 1984	*Protoribates lophothrichus* (Berlese, 1904)	+	+	+++		
	*Protoribates oblongus* (Ewing, 1909)		+	+		
Punctoribatidae Thor, 1937	*Punctoribates insignis* (Berlese, 1910)		+			
Tectocepheidae Grandjean, 1954	*Tectocepheus velatus* (Michael, 1880)	+++	++	++	++	+
Tenuialidae Jacot, 1929	*Hafenrefferia acuta* (Aoki, 1966)	++	++	++	+	++
Trhypochthoniidae Willmann, 1931	*Trhypochthonius tectorum* (Berlese, 1896)	++	+			
Xenillidae Woolley et Higgins, 1966	*Xenillus tegeocranus* (Hermann, 1804)	+				

**FIGURE 2:**
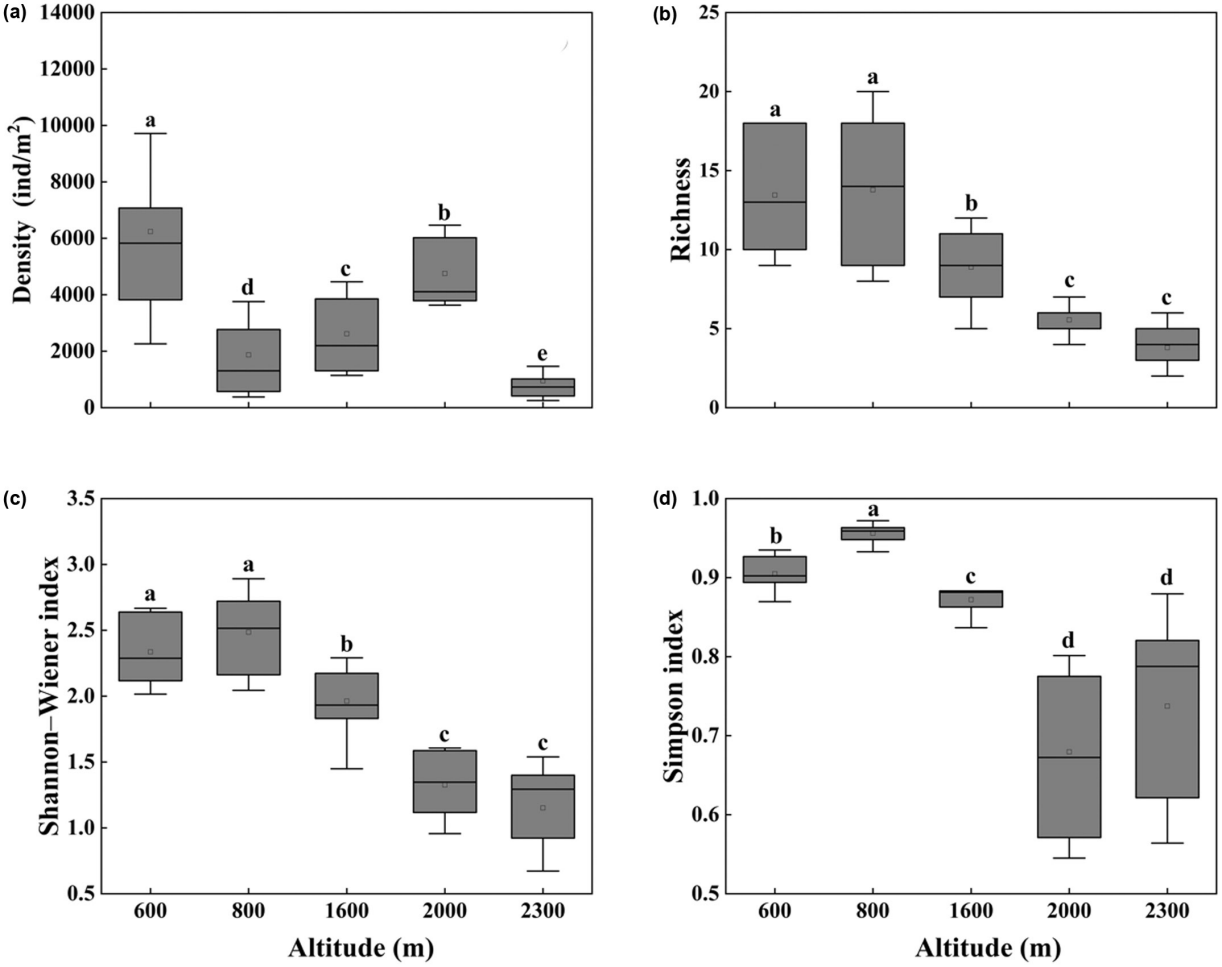
Mean density (a), species richness (b), Shannon–Wiener index (c), and Simpson index (d) of soil surface‐dwelling Oribatida assemblages along an altitudinal gradient on the Changbai Mountain, Northeast China. Data were averaged values (with standard errors). Different letters above boxes indicate significant differences between altitudes (*p* < .05, Kruskal–Wallis test).

**FIGURE 3:**
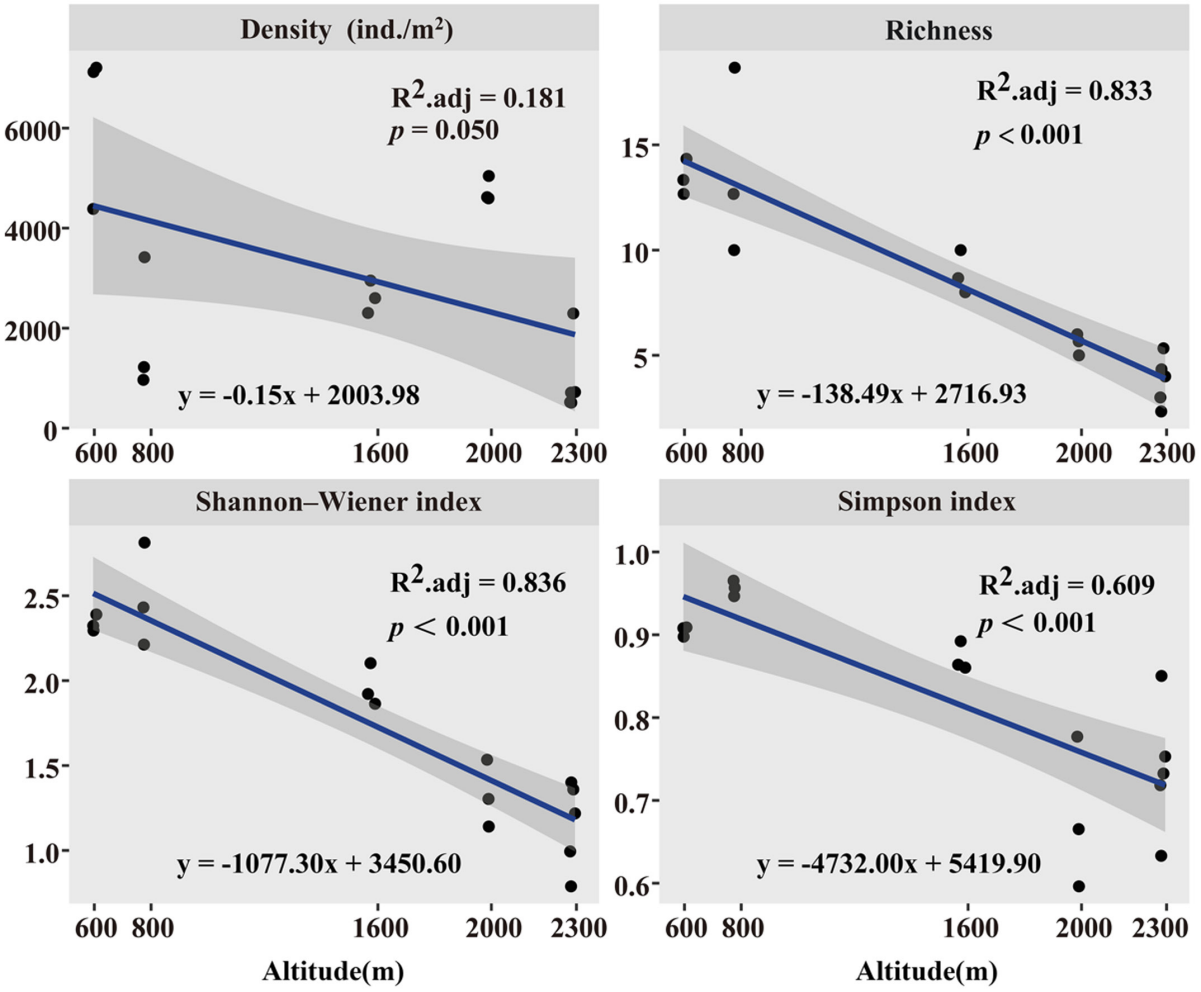
Multiple linear regressions between altitude and density, richness, Shannon–Wiener index, and Simpson index of soil surface‐dwelling Oribatida along an altitudinal gradient on the Changbai Mountain, Northeast China.

The average density of soil surface‐dwelling Oribatida was 3286.07 ± 528.44 ind./m^2^. Species richness and density were significantly different along the altitudinal gradient (Kruskal–Wallis test, *p* < .001; Figure 2a,b). Shannon–Wiener and Simpson indexes were significantly higher at 800 m than at other altitudes (Kruskal–Wallis test, *p* < .001; Figure 2c,d). The density and alpha diversity of soil surface‐dwelling Oribatida community showed a significantly negative correlation with altitude (Figure 3).

### 3.2 Beta diversity

**FIGURE 4:**
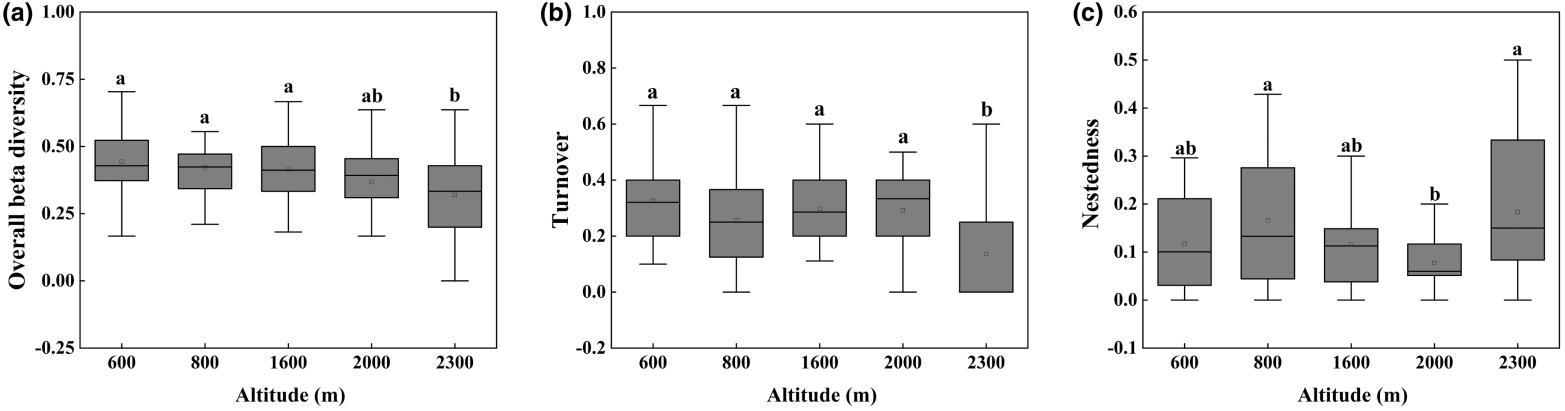
Beta diversity (a) and its turnover (b) and nestedness (c) components based on Sørensen dissimilarity between five altitudes. Different letters above boxes indicate significant differences between altitudes (*p* < .05, Kruskal–Wallis test).

Beta diversity of soil surface‐dwelling Oribatida was significantly lower at 2300 m than at other altitudes (*p* < .001, Figure 4). The partition in beta diversity revealed that differences in species composition were generally induced by species turnover and not nestedness. The 600 m, 800 m, 1600 m, and 2000 m altitudes were significantly influenced by the species turnover component, and the 2300 m altitude was significantly influenced by the species nestedness component.

### 3.3 Community assemblages

**FIGURE 5:**
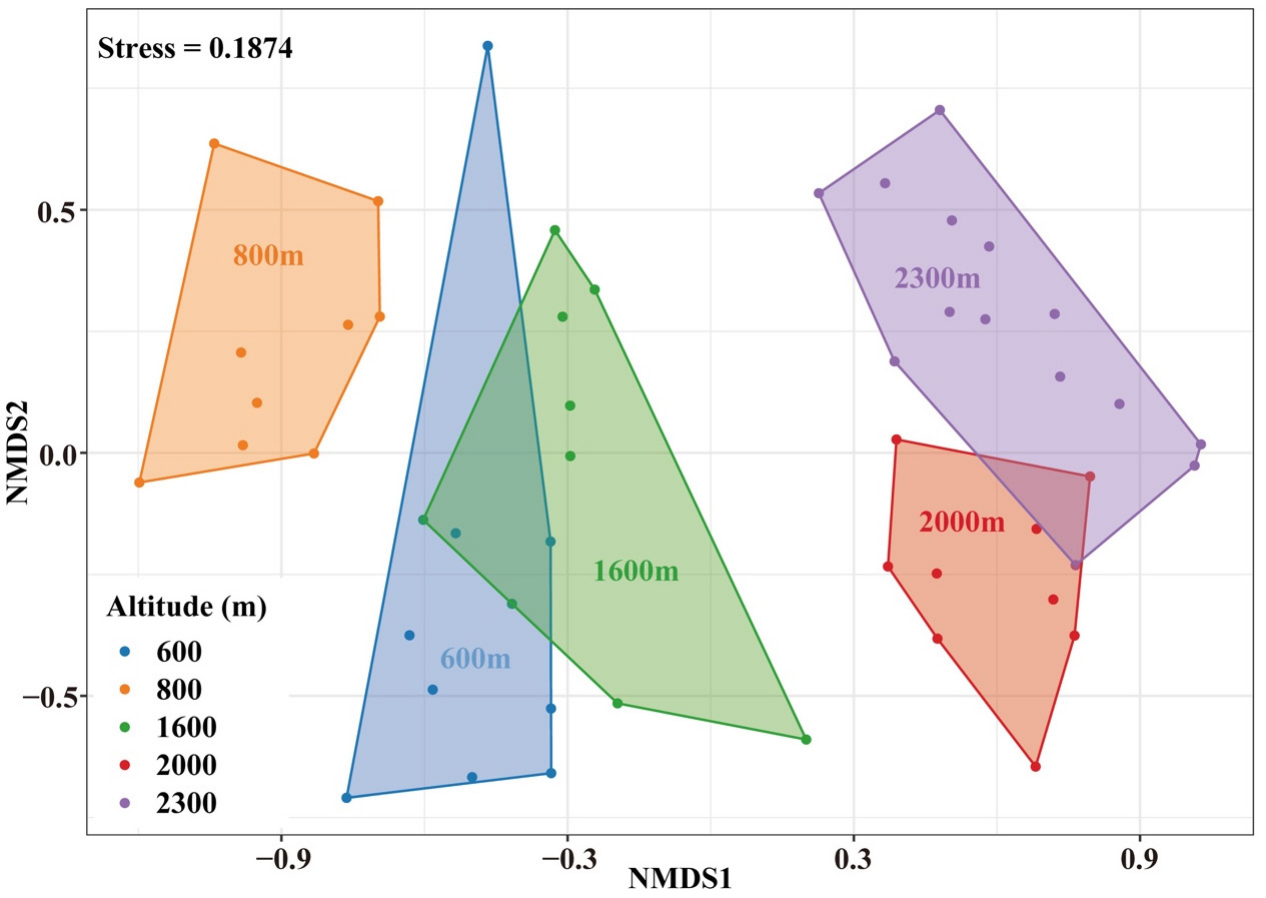
Nonmetric multidimensional scaling (NMDS) ordinations based on the Bray–Curtis dissimilarity show the soil surface‐dwelling Oribatida community assemblages along an altitudinal gradient on the Changbai Mountain, Northeast China. Each point represents the community composition of soil surface‐dwelling Oribatida in a single sample.

The non‐metric multidimensional scaling (NMDS) ordination identified five groups of the soil surface‐dwelling Oribatida (Figure 5, Stress = 0.1874, ANOSIM: *R* = .815, *p* = .001). One‐way ANOSIM showed that Oribatida assemblages significantly differed along the altitudinal gradient (Table 3). Indicator analysis revealed representative taxa for each altitude gradient (Table 4). Eight species were found as indicators at low altitudes of 600 and 800 m. *Protoribates lophothrichus* was the only indicator at 1600 m, and *Eremaeus borealis* was the only indicator at 2000 m. No indicator species were identified for the alpine tundra at 2300 m.

**TABLE 3:** Correlation analysis of one‐way ANOSIM test for composition variation of soil surface‐dwelling Oribatida assemblages along the altitudinal gradient in the Changbai Mountain, Northeast China.

	600 m	800 m	1600 m	2000 m
800 m	0.7668*			
1600 m	0.7833*	0.6096*		
2000 m	0.8608*	0.8678*	0.9757*	
2300 m	0.8646*	0.7025*	0.8298*	0.8279*

* *p* < .01.

**TABLE 4:** Indicator species among the altitudes in the Changbai Mountain, Northeast China (*p* < .05).

Indicator species	Altitude	Indicator value	*p* value
*Oribatula tibialis*	600 m	99.1	.001
*Cepheus cepheiformis*	600 m	76.8	.001
*Eupelops contaminatus*	600 m	74.5	.001
*Trhypochthonius tectorum*	600 m	71.3	<.001
*Acrotritia hauseri*	800 m	87.0	.001
*Gustavia microcephala*	800 m	81.6	.001
*Acrogalumna* sp.	800 m	62.4	.006
*Punctoribates insignis*	800 m	57.7	.016
*Protoribates lophothrichus*	1600 m	86.5	.001
*Eremaeus borealis*	2000 m	77.8	.001

### 3.4 Variation of community

**FIGURE 6:**
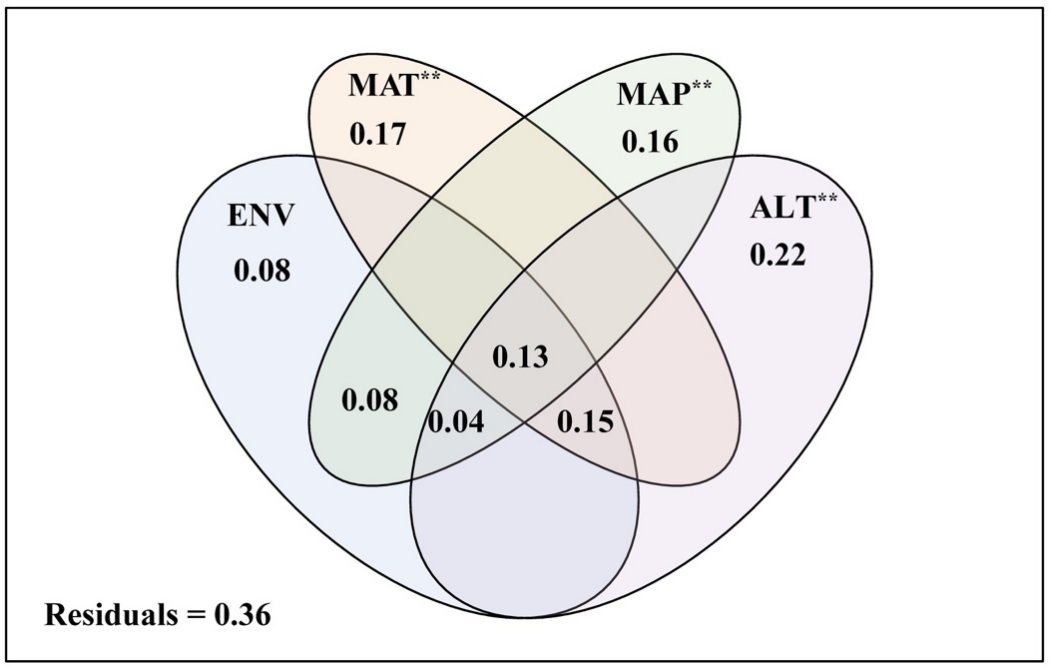
The contribution of abiotic parameters to soil surface‐dwelling Oribatida abundance along an altitudinal gradient on the Changbai Mountain, Northeast China (ALT, altitude; ENV, environmental factors including total carbon, total nitrogen, total hydrogen, total sulfur, total phosphorus, and the litter quality of per unit area; MAP, mean annual precipitation; MAT, mean annual temperature), **p < .01.

Nine factors were selected as the drivers of variation in the Oribatida community structure (Table 1). Venn diagram showed that elevation factor (altitude) and climate factors (including MAT and MAP) had an extremely significant correlation on the soil surface‐dwelling Oribatida community (Figure 6, *p* < .01). The most substantial contribution was attributed to the elevation factor (22%).

**FIGURE 7:**
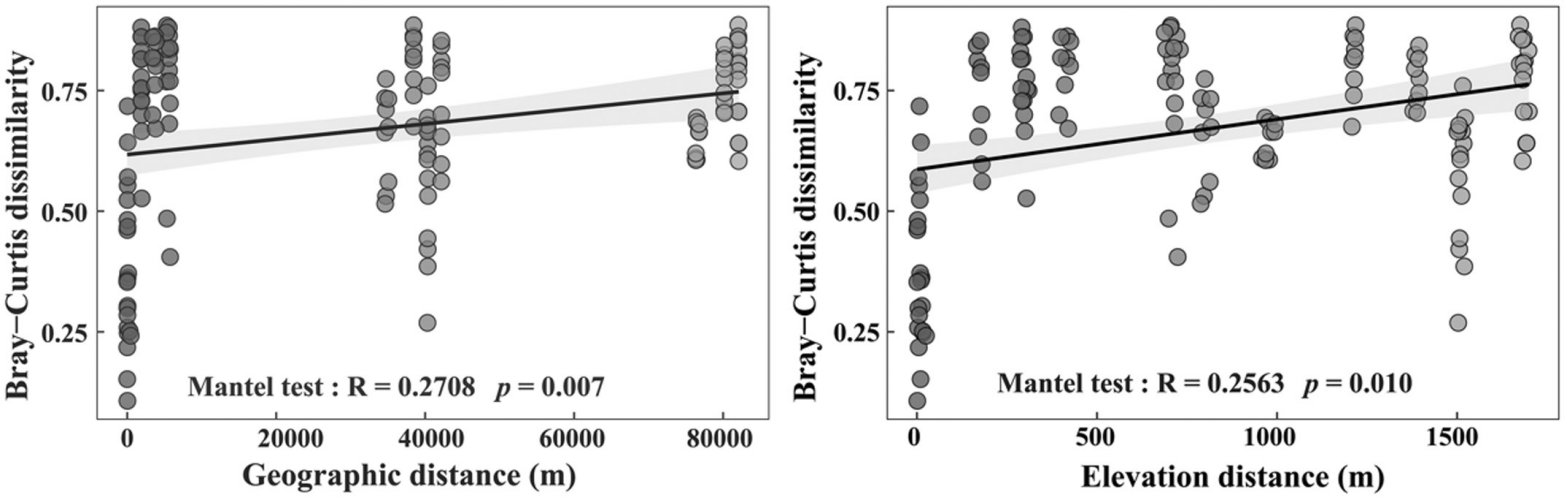
Distance–decay relationship between Oribatida community dissimilarity based on Bray–Curtis distance, with geographic and elevation distance based on their Spearman's rank correlation.

Soil surface‐dwelling Oribatida community dissimilarity had a positive correlation with geographic and elevation distance, explaining 27.08% and 25.63% of the variations, respectively (Figure 7). The variation of soil surface‐dwelling Oribatida community structure between 600 and 800 m and between 600 and 2300 m had a significant correlation with geographic distance (Table 5). The variation in soil surface‐dwelling Oribatida community structure between 1600, 2000, and 2300 m significantly correlated with elevation distance.

**TABLE 5:** The Mantel test of soil surface‐dwelling Oribatida community dissimilarity on the Changbai Mountain on geographic distances (GEO) and elevation distances (ELE).

Altitude	600 m		800 m		1600 m		2000 m	
	*r*	*p*	*r*	*p*	*r*	*p*	*r*	*p*
800 m								
GEO	.9786	.0014**						
ELE	.5201	.1056						
1600 m								
GEO	.8500	.0083**	.5321	.0819				
ELE	.7429	.0444*	.6524	.0333*				
2000 m								
GEO	.9071	.0042**	.7750	.0431*	.7714	.0403*		
ELE	.7996	.0306*	.8740	.0153*	.7750	.0389*		
2300 m								
GEO	.6431	.0180*	.5222	.0146*	.5676	.0134*	.6245	.0195*
ELE	.5980	.0206*	.3808	.0349*	.5605	.0179*	.6585	.0165*

* *p* < .05

** *p* < .01.

## 4 DISCUSSION

Our study assessed the patterns and drivers of soil surface‐dwelling Oribatida diversity along an altitudinal gradient on the Changbai Mountain. We found that the distribution pattern of alpha diversity and beta diversity of soil surface‐dwelling Oribatida decreased with the rising altitude. Species turnover was the essential component in driving beta diversity. NMDS analysis showed that the community structure of soil surface‐dwelling Oribatida assemblages varied significantly along the altitudinal gradient. The community variation of soil surface‐dwelling Oribatida was affected by altitude and climate (temperature and precipitation). Geographical and elevation distances were the primary drivers of variation in the distribution patterns of soil surface‐dwelling Oribatida communities.

### 4.1 Alpha diversity

The alpha diversity of soil surface‐dwelling Oribatida assemblages showed a decreasing trend with the rising altitude. However, another peak of soil surface‐dwelling Oribatida density was recorded at 2000 m rather than middle altitude. In our study, the sampling sites in birch forests are located at a middle‐high altitude of approximately 2000 m. A balance has been established between increased precipitation and decreased temperature, and relatively high‐quality litter per unit area had provided a diverse microhabitat for soil surface‐dwelling Oribatida (Hasegawa et al., 2006; Xu et al., 2020). Such a suitable habitat makes three species of soil surface‐dwelling Oribatida (*E. borealis*, *P. peltifer*, *T. berlesei*) reach an extremely high density, resulting in another density peak at 2000 m. This distribution pattern can also relate to edge effects, as the forest line is the most prominent biogeographic boundary of the montane ecosystem (Becker et al., 2007). The sampling area of 2000 m is a patchy transition zone consisting of several transitional vegetation zones and their components. It is near the timberline between the Changbai Mountain forest zone and the alpine tundra. Such habitat boundary often shows a high plant diversity, biomass, species dispersal, and soil nutrient resources (Reinmann & Hutyra, 2017; Ruwanza, 2018; Shen et al., 2021). For instance, compared to other vegetation zones, the microbial biomass in the Changbai Mountain forest transition zone was higher (Liu et al., 2019).

The environmental filters generally become more robust with an increased altitude (Hoiss et al., 2012; Webb et al., 2002; Xie, Chen, et al., 2022; Xie, Sun, et al., 2022). The soil surface‐dwelling Oribatida were dominated by a combination of significantly highest nestedness at 2300 m due to lack of resources, harsh climatic conditions, and topographic barriers (Castro et al., 2019; Melo et al., 2009). In our study, the 2300 m (tundra zone) altitude was dominated by two soil surface‐dwelling Oribatida species, *P. peltifer* and *T. berlesei*, which accounted for 89.72% of the total community density, associated with the loss of species occurred in the high altitude (Willig & Presley, 2016). *P. peltifer* and *T. berlesei* were not the dominant species at 600, 800, and 1600 m because a common species widely distributed at high altitudes could usually only be active in some small extreme patches in a lower altitude (Otto & Svensson, 1982).

In our study, we observed an average density of 3286 ind./m^2^ for soil surface‐dwelling Oribatida, which is considerably lower than the density of soil‐dwelling Oribatida reported by Hasegawa et al. (2006) and Illig et al. (2010), ranging from 15,000 to 25,000 ind./m^2^. The sampling tool had a large impact on experimental results (Jing et al., 2005). We compared with Liu et al. (2023) and discovered that a proportion of species of Oribatida were exclusively collected using the Vortis arthropod suction sampler, such as *Scapheremaeus polysetosus*, *Archoplophora rostralis*, *Micreremus brevipes* and *Oribotritia gigas*, and several Oribatida were exclusively collected using Tullgren extractors, such as *Parhypochthonius aphidinus*, *Liochthonius sellnicki* and *Synchthonius elegans*. Therefore, combining with different sampling tools is a better option in fieldwork, for a more accurate and comprehensive collection of sample data.

### 4.2 Beta diversity

From 800 to 2300 m, the overall beta diversity of soil surface‐dwelling Oribatida decreased gradually. A significant diversity variation of soil surface‐dwelling Oribatida occurred at 2300 m, which also supported our first Hypothesis. The overall beta diversity was significantly higher at 600, 800, and 1600 m than at other altitudes. In such cases, even a slight increase in the area could incorporate many new species of soil surface‐dwelling Oribatida (Jankowski et al., 2009). The species turnover component was the primary process of soil surface‐dwelling Oribatida community structuring, which supported our second Hypothesis. This pattern of variation dominated by species turnover reflects a deterministic process of community construction, consistent with the study by Bishop et al. (2015).

Some species have a remarkable dispersal capacity and can tolerate the frequent extreme climates and low availability of resources in high altitudes (Hasegawa et al., 2006; Illig et al., 2010; Otto & Svensson, 1982; Valencia‐Cuevas et al., 2020). In our study, some soil surface‐dwelling Oribatida (*Achipteria coleoptrata*, *Hafenrefferia acuta*, *Lauroppia neerlandica*, among others) could establish at all altitudes. In contrast, other species with narrower ecological ranges can only present at lower altitudes. This pattern reflected the substantial turnover of soil surface‐dwelling Oribatida (Marian et al., 2018). Because of the adaptive divergence of species along the altitudinal gradient, species living in low altitudes lack the adaption mechanism for middle‐high altitudes (Keller et al., 2013; Samson et al., 1997).

### 4.3 Variation of community

In our study, spatial filtering strongly correlated with the soil surface‐dwelling Oribatida community, consistent with our third Hypothesis. The elevation is a vital compound variable in montane ecosystems (Mumladze et al., 2017). We found that the elevation had an extremely significant effect on the community structure of soil surface‐dwelling Oribatida. However, other studies found that the elevation had no biological significance, and the variation of the microarthropod community along the altitudinal gradient was associated with either biotic or abiotic factors, such as plant community, rather than a response to altitude (Jiang et al., 2015; Nieto Peñalver et al., 2017; Valencia‐Cuevas et al., 2020).

Microarthropod communities are significantly associated with climatic factors, composition and quality of litter, soil characteristics including pH, and resource availability (Shen et al., 2013; Winkler et al., 2018; Xie, Sun, et al., 2022). Our study revealed that soil surface‐dwelling Oribatida were not significantly influenced by environmental factors, except for temperature and precipitation. Temperature is an indirect measure of primary productivity and a determinant of arthropod community composition, representing the altitudinal limit of species distribution in the montane ecosystem (Kaspari et al., 2000; Sundqvist et al., 2013). Studies from other ecosystems have also demonstrated the effect of precipitation on Oribatida (Hense, 2016; Lehmitz et al., 2020). Our study can support the third Hypothesis that temperature and precipitation could be the primary drivers of soil surface‐dwelling Oribatida variation along the altitudinal gradient. Microarthropod diversity is closely associated with carbon content and carbon release (Fujii et al., 2018; Liu et al., 2019), and the nitrogen content can impact the feeding behavior of microarthropods by limiting microbial communities (Devetter et al., 2017). However, the total carbon and total nitrogen content did not significantly affect the abundance of microarthropods in our study.

## 5 CONCLUSIONS

In conclusion, our results revealed a dynamic pattern of soil surface‐dwelling Oribatida diversity and community structure in montane ecosystems along altitudinal gradients. The abundance of soil surface‐dwelling Oribatida can be influenced by elevation and climate factors. Spatial factors, including geographic and elevation distance, were the primary factors associated with the variations of soil surface‐dwelling Oribatida community in montane ecosystems. Our study provides evidence for the biodiversity analyzing of soil surface‐dwelling Oribatida in montane ecosystems along an altitudinal gradient.

## AUTHOR CONTRIBUTIONS

**Yiling Lin:** Conceptualization (equal); investigation (equal); methodology (equal); writing – original draft (lead); writing – review and editing (equal). **Haitao Wu:** Conceptualization (equal); funding acquisition (lead); supervision (lead); writing – review and editing (equal). **Dong Liu:** Investigation (equal); methodology (equal). **Yaxiao Li:** Investigation (equal). **Yujuan Kang:** Investigation (equal). **Zhongsheng Zhang:** Writing – review and editing (equal). **Wenfeng Wang:** Writing – review and editing (equal).

## CONFLICT OF INTEREST STATEMENT

The authors declared that they have no conflicts of interest in this work.

## Data Availability

The environmental factors data and coordinates of sampling sites are provided in Table 1. The species data supporting the findings of this study will be openly available in Dryad: https://doi.org/10.5061/dryad.v15dv4212.

## References

[ece310105-bib-0001] Barry, R. G. (1992). Mountain climatology and past and potential future climatic changes in mountain regions: a review. Mountain Research and Development, 12, 71–86.

[ece310105-bib-0002] Baselga, A. (2010). Partitioning the turnover and nestedness components of beta diversity. Global Ecology and Biogeography, 19, 134–143.

[ece310105-bib-0003] Becker, A. , Körner, C. , Brun, J.‐J. , Guisan, A. , & Tappeiner, U. (2007). Ecological and land use studies along elevational gradients. Mountain Research and Development, 27, 58–65.

[ece310105-bib-0004] Bishop, T. R. , Robertson, M. P. , Van Rensburg, B. J. , & Parr, C. L. (2015). Contrasting species and functional beta diversity in montane ant assemblages. Journal of Biogeography, 42, 1776–1786. 27563167 10.1111/jbi.12537 PMC4979679

[ece310105-bib-0005] Bohan, D. A. , Caron‐Lormier, G. , Muggleton, S. , Raybould, A. , & Tamaddoni‐Nezhad, A. (2011). Automated discovery of food webs from ecological data using logic‐based machine learning. PLoS One, 6, e29028. 22242111 10.1371/journal.pone.0029028 PMC3248413

[ece310105-bib-0006] Bradford, M. A. , Tordoff, G. M. , Eggers, T. , Jones, T. H. , & Newington, J. E. (2002). Microbiota, fauna, and mesh size interactions in litter decomposition. Oikos, 99, 317–323.

[ece310105-bib-0007] Brühl, C. A. , Mohamed, M. , & Linsenmair, K. E. (1999). Altitudinal distribution of leaf litter ants along a transect in primary forests on mount Kinabalu, Sabah, Malaysia. Journal of Tropical Ecology, 15, 265–277.

[ece310105-bib-0008] Cáceres, M. D. , & Legendre, P. (2009). Associations between species and groups of sites: Indices and statistical inference. Ecology, 90, 3566–3574. 20120823 10.1890/08-1823.1

[ece310105-bib-0009] Castro, D. M. , Callisto, M. , Solar, R. R. , Macedo, D. R. , & Fernandes, G. W. (2019). Beta diversity of aquatic invertebrates increases along an altitudinal gradient in a Neotropical mountain. Biotropica, 51, 399–411.

[ece310105-bib-0010] Chen, L. , Wu, S. , & Pan, T. (2011). Variability of climate–growth relationships along an elevation gradient in the Changbai Mountain, northeastern China. Trees, 25, 1133–1139.

[ece310105-bib-0011] Devetter, M. , Háněl, L. , Řeháková, K. , & Doležal, J. (2017). Diversity and feeding strategies of soil microfauna along elevation gradients in Himalayan cold deserts. PLoS One, 12, e0187646. 29131839 10.1371/journal.pone.0187646 PMC5683576

[ece310105-bib-0012] Dufrêne, M. , & Legendre, P. (1997). Species assemblages and indicator species: The need for a flexible asymmetrical approach. Ecological Monographs, 67, 345–366.

[ece310105-bib-0013] Fick, S. E. , & Hijmans, R. J. (2017). WorldClim 2: New 1‐km spatial resolution climate surfaces for global land areas. International Journal of Climatology, 37, 4302–4315.

[ece310105-bib-0014] Fitzgerald, J. , & Solomon, M. (2004). Can flowering plants enhance numbers of beneficial arthropods in UK apple and pear orchards? Biocontrol Science and Technology, 14, 291–300.

[ece310105-bib-0015] Freeman, B. G. (2020). Lower elevation animal species do not tend to be better competitors than their higher elevation relatives. Global Ecology and Biogeography, 29, 171–181.

[ece310105-bib-0016] Fujii, S. , Cornelissen, J. H. , Berg, M. P. , & Mori, A. S. (2018). Tree leaf and root traits mediate soil faunal contribution to litter decomposition across an elevational gradient. Functional Ecology, 32, 840–852.

[ece310105-bib-0017] García‐Navas, V. , Sattler, T. , Schmid, H. , & Ozgul, A. (2020). Temporal homogenization of functional and beta diversity in bird communities of the Swiss Alps. Diversity and Distributions, 26, 900–911.

[ece310105-bib-0018] Gaston, K. J. (2000). Global patterns in biodiversity. Nature, 405, 220–227. 10821282 10.1038/35012228

[ece310105-bib-0019] Gentili, R. , Armiraglio, S. , Sgorbati, S. , & Baroni, C. (2013). Geomorphological disturbance affects ecological driving forces and plant turnover along an altitudinal stress gradient on alpine slopes. Plant Ecology, 214, 571–586.

[ece310105-bib-0020] González, G. , & Seastedt, T. R. (2001). Soil fauna and plant litter decomposition in tropical and subalpine forests. Ecology, 82, 955–964.

[ece310105-bib-0021] Hasegawa, M. , Ito, M. , & Kitayama, K. (2006). Community structure of oribatid mites in relation to elevation and geology on the slope of mount Kinabalu, Sabah, Malaysia. European Journal of Soil Biology, 42, S191–S196.

[ece310105-bib-0022] Heino, J. , Melo, A. S. , Siqueira, T. , Soininen, J. , Valanko, S. , & Bini, L. M. (2015). Metacommunity organisation, spatial extent and dispersal in aquatic systems: Patterns, processes and prospects. Freshwater Biology, 60, 845–869.

[ece310105-bib-0023] Hense, J. B. (2016). Oribatid mite (Acari: Oribatida) and Chironomid (Diptera: Chironomidae) communities from a high‐Andean cushion peatland in Peru (14S) and their use for palaeoenvironmental reconstruction during the Nasca cultural period. Universitäts‐und Stadtbibliothek Köln.

[ece310105-bib-0024] Hijmans, R. J. , Williams, E. , & Vennes, C. (2019). Geosphere: Spherical trigonometry. R Package Version, 1.

[ece310105-bib-0025] Hoiss, B. , Krauss, J. , Potts, S. G. , Roberts, S. , & Steffan‐Dewenter, I. (2012). Altitude acts as an environmental filter on phylogenetic composition, traits and diversity in bee communities. Proceedings of the Royal Society B: Biological Sciences, 279, 4447–4456. 10.1098/rspb.2012.1581 PMC3479805 22933374

[ece310105-bib-0026] Huhta, V. (2007). The role of soil fauna in ecosystems: A historical review. Pedobiologia, 50, 489–495.

[ece310105-bib-0027] Illig, J. , Norton, R. A. , Scheu, S. , & Maraun, M. (2010). Density and community structure of soil‐and bark‐dwelling microarthropods along an altitudinal gradient in a tropical montane rainforest. Experimental and Applied Acarology, 52, 49–62. 20229099 10.1007/s10493-010-9348-x PMC2914295

[ece310105-bib-0028] Jankowski, J. E. , Ciecka, A. L. , Meyer, N. Y. , & Rabenold, K. N. (2009). Beta diversity along environmental gradients: Implications of habitat specialization in tropical montane landscapes. Journal of Animal Ecology, 78, 315–327. 19040686 10.1111/j.1365-2656.2008.01487.x

[ece310105-bib-0029] Jiang, Y. , Xiuqin, Y. , & Fubin, W. (2015). Composition and spatial distribution of soil mesofauna along an elevation gradient on the north slope of the Changbai Mountains, China. Pedosphere, 25, 811–824.

[ece310105-bib-0030] Jing, S. , Solhøy, T. , Huifu, W. , Vollan, T. I. , & Rumei, X. (2005). Differences in soil arthropod communities along a high altitude gradient at Shergyla Mountain, Tibet, China. Arctic, Antarctic, and Alpine Research, 37, 261–266.

[ece310105-bib-0031] Kaspari, M. , Odonnell, S. , & Kercher, J. R. (2000). Energy, density, and constraints to species richness: Ant assemblages along a productivity gradient. The American Naturalist, 155, 280–293. 10.1086/303313 10686166

[ece310105-bib-0032] Keller, I. , Alexander, J. M. , Holderegger, R. , & Edwards, P. J. (2013). Widespread phenotypic and genetic divergence along altitudinal gradients in animals. Journal of Evolutionary Biology, 26, 2527–2543. 24128377 10.1111/jeb.12255

[ece310105-bib-0033] Koleff, P. , Gaston, K. J. , & Lennon, J. J. (2003). Measuring beta diversity for presence–absence data. Journal of Animal Ecology, 72, 367–382.

[ece310105-bib-0034] Legendre, P. , & Legendre, L. (2012). Numerical ecology. Elsevier.

[ece310105-bib-0035] Lehmitz, R. , Haase, H. , Otte, V. , & Russell, D. (2020). Bioindication in peatlands by means of multi‐taxa indicators (Oribatida, Araneae, Carabidae, vegetation). Ecological Indicators, 109, 105837.

[ece310105-bib-0036] Li, Z. , Jiang, X. , Wang, J. , Meng, X. , Heino, J. , & Xie, Z. (2019). Multiple facets of stream macroinvertebrate alpha diversity are driven by different ecological factors across an extensive altitudinal gradient. Ecology and Evolution, 9, 1306–1322. 30805161 10.1002/ece3.4841 PMC6374682

[ece310105-bib-0037] Liu, D. , Liu, D. , Yu, H. , & Wu, H. (2023). Strong variations and shifting mechanisms of altitudal diversity and abundance patterns in soil oribatid mites (Acari: Oribatida) on the Changbai Mountain, China. Applied Soil Ecology, 186, 104808.

[ece310105-bib-0038] Liu, Y. , Wang, L. , He, R. , Chen, Y. , Xu, Z. , Tan, B. , Zhang, L. , Xiao, J. , Zhu, P. , & Chen, L. (2019). Higher soil fauna abundance accelerates litter carbon release across an alpine forest‐tundra ecotone. Scientific Reports, 9, 1–12. 31332217 10.1038/s41598-019-47072-0 PMC6646657

[ece310105-bib-0039] Lussenhop, J. , & Bassirirad, H. (2005). Collembola effects on plant mass and nitrogen acquisition by ash seedlings (Fraxinus pennsylvanica). Soil Biology and Biochemistry, 37, 645–650.

[ece310105-bib-0040] Marian, F. , Sandmann, D. , Krashevska, V. , Maraun, M. , & Scheu, S. (2018). Altitude and decomposition stage rather than litter origin structure soil microarthropod communities in tropical montane rainforests. Soil Biology and Biochemistry, 125, 263–274.

[ece310105-bib-0041] Mayor, J. R. , Sanders, N. J. , Classen, A. T. , Bardgett, R. D. , Clement, J.‐C. , Fajardo, A. , Lavorel, S. , Sundqvist, M. K. , Bahn, M. , & Chisholm, C. (2017). Elevation alters ecosystem properties across temperate treelines globally. Nature, 542, 91–95. 28117440 10.1038/nature21027

[ece310105-bib-0042] Melo, A. S. , Rangel, T. F. L. , & Diniz‐Filho, J. (2009). Environmental drivers of beta‐diversity patterns in new‐world birds and mammals. Ecography, 32, 226–236.

[ece310105-bib-0043] Miller, G. T. , & Spoolman, S. (2011). Living in the environment: Principles, connections, and solutions. Cengage Learning.

[ece310105-bib-0044] Mumladze, L. , Murvanidze, M. , & Maraun, M. (2017). Patterns of oribatid mite species diversity: Testing the effects of elevation, area and sampling effort. Experimental and Applied Acarology, 72, 245–262. 28717996 10.1007/s10493-017-0153-7

[ece310105-bib-0045] Mumladze, L. , Murvanidze, M. , Maraun, M. , & Salakaia, M. (2015). Oribatid mite communities along an elevational gradient in Sairme gorge (Caucasus). Experimental and Applied Acarology, 66, 41–51. 25761919 10.1007/s10493-015-9893-4

[ece310105-bib-0046] Niedbała, W. , & Liu, D. (2018). Catalogue of ptyctimous mites (Acari, Oribatida) of the world. Zootaxa, 4393, 1–238. 29690402 10.11646/zootaxa.4393.1.1

[ece310105-bib-0047] Nieto Peñalver, M. C. , Dos Santos, D. A. , Izquierdo, A. E. , Rodríguez, J. S. , & Grau, H. R. (2017). Modelling beta diversity of aquatic macroinvertebrates in high Andean wetlands. Journal of Limnology, 76, 555–570.

[ece310105-bib-0048] Odland, A. (2009). Interpretation of altitudinal gradients in south Central Norway based on vascular plants as environmental indicators. Ecological Indicators, 9, 409–421.

[ece310105-bib-0049] Oksanen, J. , Blanchet, F. , Kindt, R. , Legendre, P. , Minchin, P. , O'hara, R. , Simpson, G. , Solymos, P. , Stevens, M. , & Wagner, H. (2013). Package ‘vegan’. Community Ecology Package, version 2.

[ece310105-bib-0050] Olson, D. M. (1994). The distribution of leaf litter invertebrates along a Neotropical altitudinal gradient. Journal of Tropical Ecology, 10, 129–150.

[ece310105-bib-0051] Otto, C. , & Svensson, B. S. (1982). Structure of communities of ground‐living spiders along altitudinal gradients. Ecography, 5, 35–47.

[ece310105-bib-0052] Peters, M. K. , Hemp, A. , Appelhans, T. , Behler, C. , Classen, A. , Detsch, F. , Ensslin, A. , Ferger, S. W. , Frederiksen, S. B. , & Gebert, F. (2016). Predictors of elevational biodiversity gradients change from single taxa to the multi‐taxa community level. Nature Communications, 7, 1–11. 10.1038/ncomms13736 PMC5192166 28004657

[ece310105-bib-0053] R Core Team . (2022). R: A language and environment for statistical computing. R Foundation for Statistical Computing.

[ece310105-bib-0054] Rahbek, C. , Borregaard, M. K. , Antonelli, A. , Colwell, R. K. , Holt, B. G. , Nogues‐Bravo, D. , Rasmussen, C. M. Ø. , Richardson, K. , Rosing, M. T. , Whittaker, R. J. , & Fjeldså, J. (2019). Building mountain biodiversity: Geological and evolutionary processes. Science, 365, 1114–1119. 31515384 10.1126/science.aax0151

[ece310105-bib-0055] Reinmann, A. B. , & Hutyra, L. R. (2017). Edge effects enhance carbon uptake and its vulnerability to climate change in temperate broadleaf forests. Proceedings of the National Academy of Sciences, 114, 107–112. 10.1073/pnas.1612369114 PMC5224393 27994137

[ece310105-bib-0056] Reusch, T. B. , & Wood, T. E. (2007). Molecular ecology of global change. Molecular Ecology, 16, 3973–3992. 17894755 10.1111/j.1365-294X.2007.03454.x

[ece310105-bib-0057] Ruwanza, S. (2018). The edge effect on plant diversity and soil properties in abandoned fields targeted for ecological restoration. Sustainability, 11, 140.

[ece310105-bib-0058] Ryabinin, N. A. , Liu, D. , Gao, M. , & Wu, D. H. (2018). Checklist of oribatid mites (Acari, Oribatida) of the Russian Far East and northeast of China. Zootaxa, 4472, 201–232. 30313366 10.11646/zootaxa.4472.2.1

[ece310105-bib-0059] Samson, D. A. , Rickart, E. A. , & Gonzales, P. C. (1997). Ant diversity and abundance along an Elevational gradient in The Philippines 1. Biotropica, 29, 349–363.

[ece310105-bib-0060] Shannon, C. E. (1948). A mathematical theory of communication. The Bell System Technical Journal, 27, 379–423.

[ece310105-bib-0061] Shao, G. (2011). Long‐term field studies of old‐growth forests on Changbai Mountain in Northeast China. Annals of Forest Science, 68, 885–887.

[ece310105-bib-0062] Sharma, N. , Behera, M. D. , Das, A. P. , & Panda, R. M. (2019). Plant richness pattern in an elevation gradient in the eastern Himalaya. Biodiversity and Conservation, 28, 2085–2104.

[ece310105-bib-0063] Shen, C. , He, J.‐Z. , & Ge, Y. (2021). Seasonal dynamics of soil microbial diversity and functions along elevations across the treeline. Science of the Total Environment, 794, 148644. 34192632 10.1016/j.scitotenv.2021.148644

[ece310105-bib-0064] Shen, C. , Xiong, J. , Zhang, H. , Feng, Y. , Lin, X. , Li, X. , Liang, W. , & Chu, H. (2013). Soil pH drives the spatial distribution of bacterial communities along elevation on Changbai Mountain. Soil Biology and Biochemistry, 57, 204–211.

[ece310105-bib-0065] Simpson, E. H. (1949). Measurement of diversity. Nature, 163, 688.

[ece310105-bib-0066] Stewart, A. J. (2002). Techniques for sampling Auchenorrhyncha in grasslands. Denisia, 4, 491–512.

[ece310105-bib-0067] Sundqvist, M. K. , Sanders, N. J. , & Wardle, D. A. (2013). Community and ecosystem responses to elevational gradients: Processes, mechanisms, and insights for global change. Annual Review of Ecology, Evolution, and Systematics, 44, 261–280.

[ece310105-bib-0068] Tan, B. , Wu, F.‐Z. , Yang, W.‐Q. , Liu, L. , & Yu, S. (2010). Characteristics of soil animal community in the subalpine/alpine forests of western Sichuan during onset of freezing. Acta Ecologica Sinica, 30, 93–99.

[ece310105-bib-0069] Valencia‐Cuevas, L. , Rodríguez‐Domínguez, A. , Mussali‐Galante, P. , Ramos‐Quintana, F. , & Tovar‐Sánchez, E. (2020). Influence of edaphic factors along an altitudinal gradient on a litter arthropod community in an Abies‐Quercus forest in Mexico. Acta Oecologica, 108, 103609.

[ece310105-bib-0070] Wang, X. , Wiegand, T. , Anderson‐Teixeira, K. J. , Bourg, N. A. , Hao, Z. , Howe, R. , Jin, G. , Orwig, D. A. , Spasojevic, M. J. , & Wang, S. (2018). Ecological drivers of spatial community dissimilarity, species replacement and species nestedness across temperate forests. Global Ecology and Biogeography, 27, 581–592.

[ece310105-bib-0071] Webb, C. O. , Ackerly, D. D. , Mcpeek, M. A. , & Donoghue, M. J. (2002). Phylogenies and community ecology. Annual Review of Ecology and Systematics, 33, 475–505.

[ece310105-bib-0072] Willig, M. R. , & Presley, S. J. (2016). Biodiversity and metacommunity structure of animals along altitudinal gradients in tropical montane forests. Journal of Tropical Ecology, 32, 421–436.

[ece310105-bib-0073] Winkler, M. , Illmer, P. , Querner, P. , Fischer, B. M. , Hofmann, K. , Lamprecht, A. , Praeg, N. , Schied, J. , Steinbauer, K. , & Pauli, H. (2018). Side by side? Vascular plant, invertebrate, and microorganism distribution patterns along an alpine to nival elevation gradient. Arctic, Antarctic, and Alpine Research, 50, e1475951.

[ece310105-bib-0074] Wu, G. , Xiao, H. , Zhao, J. , Shao, G. , & Li, J. (2002). Forest ecosystem services of Changbai Mountain in China. Science in China Series C: Life Sciences, 45, 21–32. 18763060 10.1360/02yc9003

[ece310105-bib-0075] Wu, H. , Lu, M. , Lu, X. , Guan, Q. , & He, X. (2015). Interactions between earthworms and mesofauna has no significant effect on emissions of CO_2_ and N_2_O from soil. Soil Biology and Biochemistry, 88, 294–297.

[ece310105-bib-0076] Xie, Z. , Chen, T. W. , Potapov, M. , Zhang, F. , Wu, D. , Scheu, S. , & Sun, X. (2022). Ecological and evolutionary processes shape below‐ground springtail communities along an elevational gradient. Journal of Biogeography, 49, 469–482.

[ece310105-bib-0077] Xie, Z. , Sun, X. , Lux, J. , Chen, T. W. , Potapov, M. , Wu, D. , & Scheu, S. (2022). Drivers of Collembola assemblages along an altitudinal gradient in Northeast China. Ecology and Evolution, 12, e8559. 35169449 10.1002/ece3.8559 PMC8840876

[ece310105-bib-0078] Xu, G. , Zhang, Y. , Zhang, S. , & Ma, K. (2020). Biodiversity associations of soil fauna and plants depend on plant life form and are accounted for by rare taxa along an elevational gradient. Soil Biology and Biochemistry, 140, 107640.

[ece310105-bib-0079] Yin, R. , Eisenhauer, N. , Auge, H. , Purahong, W. , Schmidt, A. , & Schaedler, M. (2019). Additive effects of experimental climate change and land use on faunal contribution to litter decomposition. Soil Biology and Biochemistry, 131, 141–148.

[ece310105-bib-0080] Yin, W. (1998). Pictorical keys to soil animals of China. Science Press.

[ece310105-bib-0081] Yu, D. , Liu, J. , Zhou, L. , Zhou, W. , Fang, X. , Wei, Y. , Jiang, S. , & Dai, L. (2013). Spatial variation and temporal instability in the climate–growth relationship of Korean pine in the Changbai Mountain region of Northeast China. Forest Ecology and Management, 300, 96–105.

[ece310105-bib-0082] Zentane, E. , Quenu, H. , Graham, R. I. , & Cherrill, A. (2016). Suction samplers for grassland invertebrates: Comparison of numbers caught using Vortis™ and G‐vac devices. Insect Conservation and Diversity, 9, 470–474.

